# Effectiveness of continuity of care in postoperative patients with cervical cancer: a systematic evaluation and meta-analysis of a randomized controlled trial

**DOI:** 10.3389/fonc.2024.1461296

**Published:** 2024-12-16

**Authors:** Wenfeng Fu, Qiannan Xu, Haizhen Lu, Yuanyuan Yang, Yanxue Zheng

**Affiliations:** Comprehensive Gynecology Ward of Affiliated Hospital of Jining Medical University, Jining, Shandong, China

**Keywords:** cervical cancer, continuity of care, randomized controlled trial, meta-analysis, nursing care

## Abstract

**Objective:**

To analyze the effect of implementing continuity of care for postoperative patients with cervical cancer, to improve the continuity of care model in China, and to provide comprehensive, continuous, and personalized care services for patients.

**Methods:**

PubMed, Web of Science, Science Direct, China Knowledge Network, Wanfang database, China Biomedical sources Service System, Wipro, Cochrane Library, Embase, and other databases were searched for relevant sources on the effect of continuity of care on postoperative cervical cancer patients. The Cochrane Handbook of Systematic Evaluation and Revman 5.3 software was used to evaluate the quality of the retrieved sources and perform meta-analysis.

**Results:**

Compared with the control group, extended care could improve patients’ quality of life [SMD=1.35,95%CI(1.05,1.64), P<0.05], alleviate patients’ postoperative anxiety [SMD=-0.92,95%CI(-1.85,0.00), P<0.05] and postoperative depression [SMD=-1.15,95% CI(-1.35,-0.95), P<0.05].

**Conclusion:**

The use of continuity of care form has an important role in improving the quality of life of cervical cancer patients and improving negative emotions.

## 1 Introduction

Cervical cancer is a common gynecological tumor and has become an important public health problem for women. According to the statistics on the incidence and mortality of female cancer patients around the world (1), cervical cancer is the fourth in the incidence of female malignant tumors, the first in the incidence of gynecological malignant tumors, and the fourth in the mortality rate of female malignant tumors, which poses a serious threat to women’s life and health. Cervical cancer is also one of the top ten malignant tumors in China. It is the second most prevalent malignant tumor among Chinese women, with 98.9/100,000 new cases per year, accounting for about 28.8% of the global new cases, and 30.5/100,000 cases (2). The number of cervical cancer survivors has increased with further advances in early screening, diagnosis, and treatment techniques (surgery, radiotherapy, chemotherapy, and immunotherapy) for cervical cancer, and continued improvements in clinical outcomes (3). Surgery is now one of the most effective treatments for cervical cancer, but 1/3 of patients still experience cancer progression or recurrence after surgery (4). Studies have found that when patients have more extensive surgery, the impact on their health status is more significant. Most patients with cervical cancer exhibit discomfort such as pain, fatigue, and poor appetite after surgical treatment (5). Bladder dysfunction, perimenopausal symptoms, sexual dysfunction, and lymphedema were reported among the long-term effects (6). Thus, radical cervical cancer surgery can have some degree of impact on patients’ physical and mental health and quality of life.

Continuity of care is a new model of care, which mainly refers to the continuation of medical care after patients return from hospitals to their families, and its original intention is to provide continuous and coordinated health guidance for patients after discharge from hospitals, to promote the physical rehabilitation of patients and to improve their quality of life (7). Continuity of care can avoid the problem of inadequate care in clinical diagnosis and treatment, prevent and reduce the occurrence of postoperative complications, improve the postoperative status of patients, save medical resources, and realize good social and economic benefits (8–10). At present, overseas countries have established a perfect continuity of care system, and have widely implemented continuity of care activities in chronic diseases such as cardiovascular disease, cancer, diabetes, etc. Strengthening post-discharge follow-up and enhancing communication and collaboration between health services, improves the clinical outcomes of patients, reduces the readmission rate of patients, saves medical costs, and improves the service recognition and quality of life of patients (11). In recent years, continuity of care in China has also developed rapidly, and a large number of continuity of care services have been carried out in patients with cardiovascular and cerebrovascular diseases, oncology chemotherapy, respiratory diseases, diabetes mellitus, amputation reimplantation, and dialysis (12, 13).

At present, continuity nursing has also been applied to a certain extent in the treatment of cervical cancer, and research in this area has relatively increased and achieved certain research results. The continuity of nursing care can greatly improve the quality of life of cervical cancer patients after surgery, and ensure the continuity, repeatability, completeness, and continuity of nursing care for cervical cancer patients after discharge from the hospital. Although scholars have conducted relevant interventional studies on continuity of care for cervical cancer patients, systematic evaluation of continuity of care is lacking. This study evaluates the meta-analysis of the impact of continuity of care on the quality of life of postoperative patients with cervical cancer to provide an evidence-based basis for clinical interventions for postoperative patients with cervical cancer and to further improve the continuity of care model in the future.

## 2 Methods

### 2.1 Sources inclusion and exclusion criteria

Inclusion criteria: ① The type of study was randomized controlled trial (RCT); ② The study subjects were patients over 18 years of age, clearly diagnosed with cervical cancer, and having undergone radical cervical cancer surgery; ③ The interventions were: the control group was the patients who took routine care; the observation group was the postoperative patients who received extended care; ④ The outcome indicators were: (1) Quality of life: the quality of life scale (SF-36) was used to assess the quality of life of the patients.(2) Anxiety: Self-rating Anxiety Scale (SAS); (3) Depression: Self-rating Depression Scale (SDS).

Exclusion criteria: (1) duplicate publication; (2) non-Chinese and English sources; (3) unavailability of original text and original data; (4) poor quality of research.

### 2.2 Search strategy

**Figure 1:**
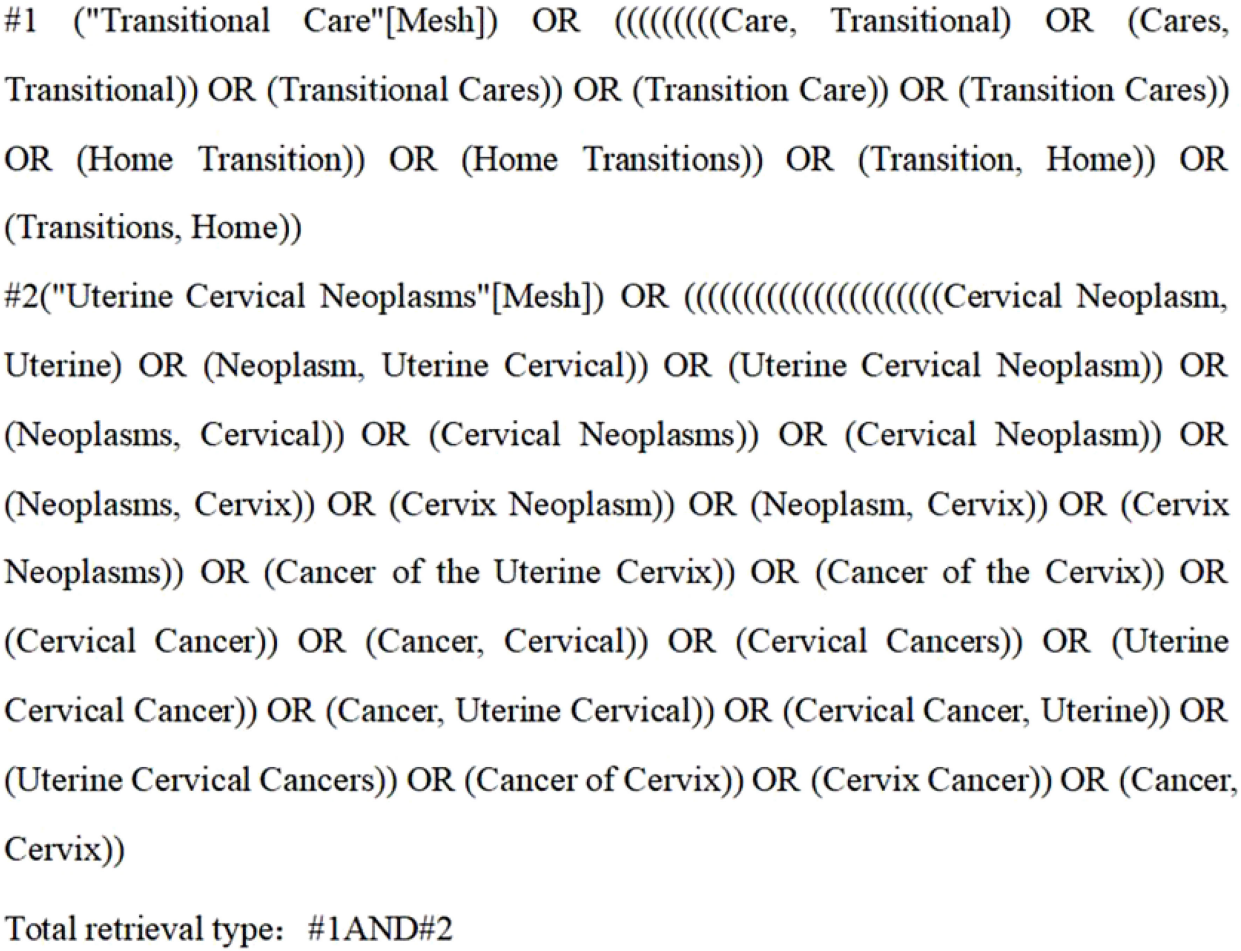
Pubmed sources search format.

Systematic search of PubMed, Web of science, science direct, China Knowledge Network, Wanfang Database, China Biomedical sources Service System, Wipro, Cochrane Library, embase and other databases for studies on the impact of continuity of care on postoperative patients with cervical cancer, with a timeframe of the construction of the database to April 2024, was performed on the relevant sources was traced. The search strategy was adapted to different databases by combining subject terms with free words. The Chinese search terms were “cervical cancer”, “cervical tumor”, “continuity of care”, “transitional care”, “extended care” “continuity of service” “continuum of care” “quality of life” “quality of survival” and so on. The English search uses a combination of subject words and free words, the search words are “uterine cervicalneoplasms”, “continuum of care”, “continuity of care”, “transitional care”, “transition of care”, “extended care”, “continuity of care”, “quality of life”, “quality of survival” and so on. care” “transition of care” “continuity of nursing” “extended care” “life quality ““health-related quality of life”. The sources search process conformed to the search requirements in the PRISMA (14) statement. pubmed’s search process was as follows (**Figure 1**).

### 2.3 Sources screening and data extraction

The sources was sorted out using Endnote X9 software with reference to the inclusion and exclusion criteria. Sources screening and data extraction were carried out independently by two subject members who had received training in evidence-based methodology and had long been involved in the care of patients with cervical cancer, and in case of disagreement, the decision was made in consultation with the third. The main extracted information included first author, year of publication, province, number of study participants, intervention method, and primary outcome indicators.

### 2.4 Sources quality assessment

The quality of the included sources was evaluated by two panelists according to the Cochrane (15) Risk of Bias Assessment Tool, and in case of disagreement, the decision was made in consultation with the third panelist. The Cochrane Risk of Bias Assessment Tool mainly includes random sequence generation, allocation concealment, selection of blinding, completeness of outcome data, selective reporting of results, and other biases, each of which was evaluated as “low risk”, “high risk”, “unclear”. “unclear” for each item. A grade of A was assigned if all of the items met the “low risk” criteria, a grade of B was assigned if some of the items met the “low risk” criteria, and a grade of C was assigned if none of the items met the “low risk” criteria.

### 2.5 Statistical methods

Excel and Review Manager 5.3 software were used to plot for statistical analysis. If the heterogeneity was small (P≥0.1,I^2^ <50%), a fixed-effects model was used; if the heterogeneity was large (P<0.1,I^2^ >50%), a random-effects model was chosen. The outcome indicators were continuous variables, and the measurement tools were different, using mean difference (MD), standardized mean difference (SMD), and 95% confidence interval (CI) to describe the continuous variables, when P ≤ 0.05 differences between the experimental and control groups were considered to exist and were statistically significant.

## 3 Results

### 3.1 Results of the sources search

**Figure 2:**
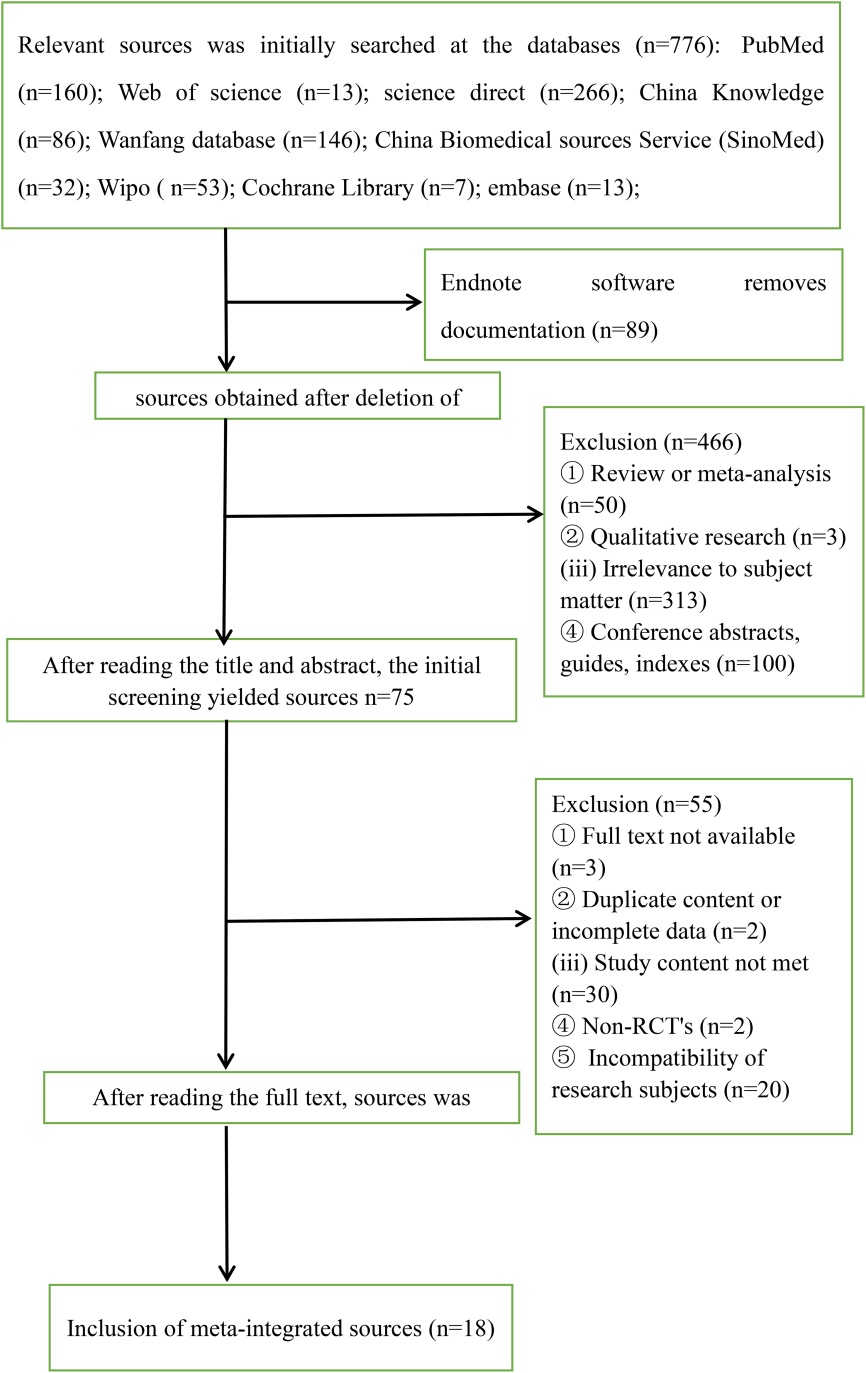
Flowchart of sources screening.

A total of 776 sources were obtained from the initial screening. After using Endnote software to eliminate 89 duplicates and manually screening 146 duplicates to obtain 541 documents, 75 documents were obtained after eliminating 50 meta-analyses, systematic evaluations, etc., three qualitative studies, 100 documents of conference abstracts, guidelines, indexes, and 313 documents that were not related to the topic. After reading through the full text and eliminating three articles that could not obtain the full text, two articles with duplicated content or incomplete data, 30 articles that did not conform to the content of the study, two articles that were not randomized controlled trials, and 20 articles that did not conform to the study subjects, 18 articles were finally included (16–33). The flowchart of sources search is shown in **Figure 2**.

### 3.2 Basic characteristics of the included sources and evaluation of methodological quality

**Figure 3:**
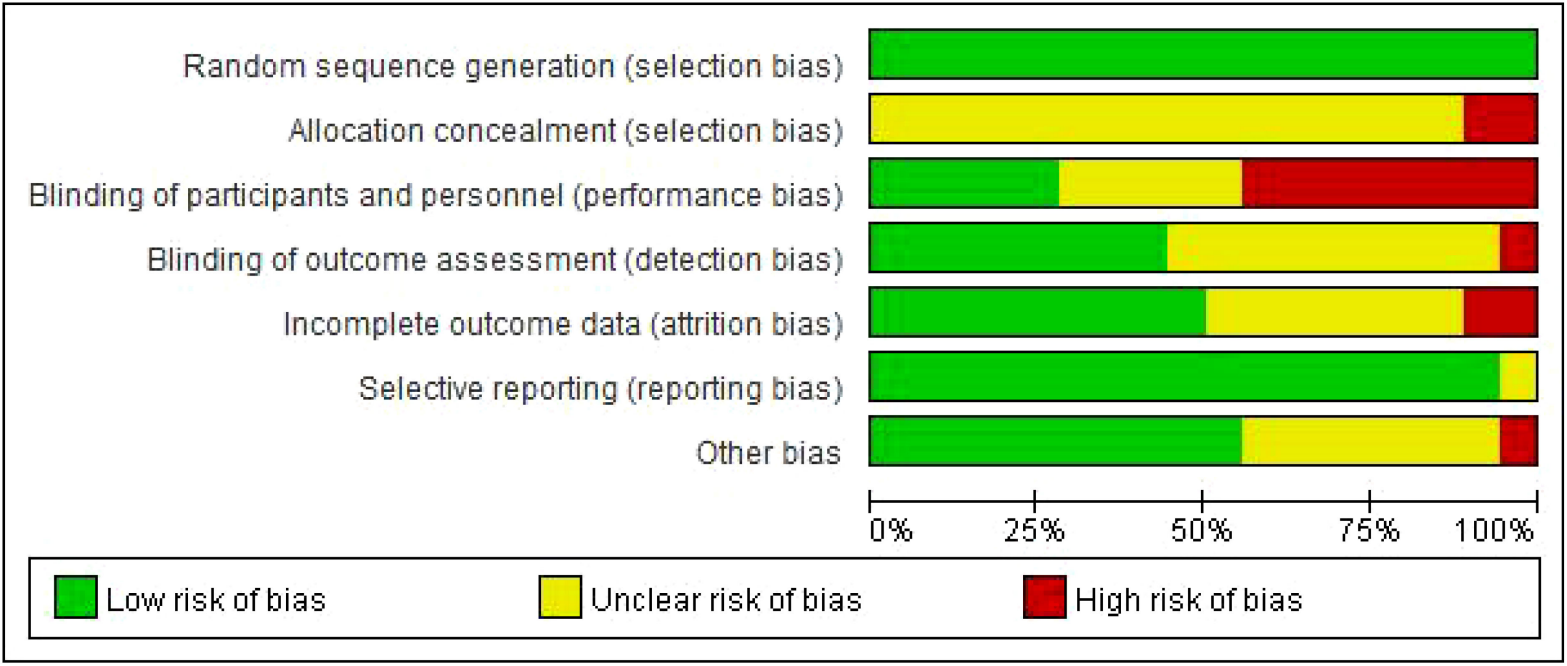
Summary of sources quality assessment.

**Figure 4:**
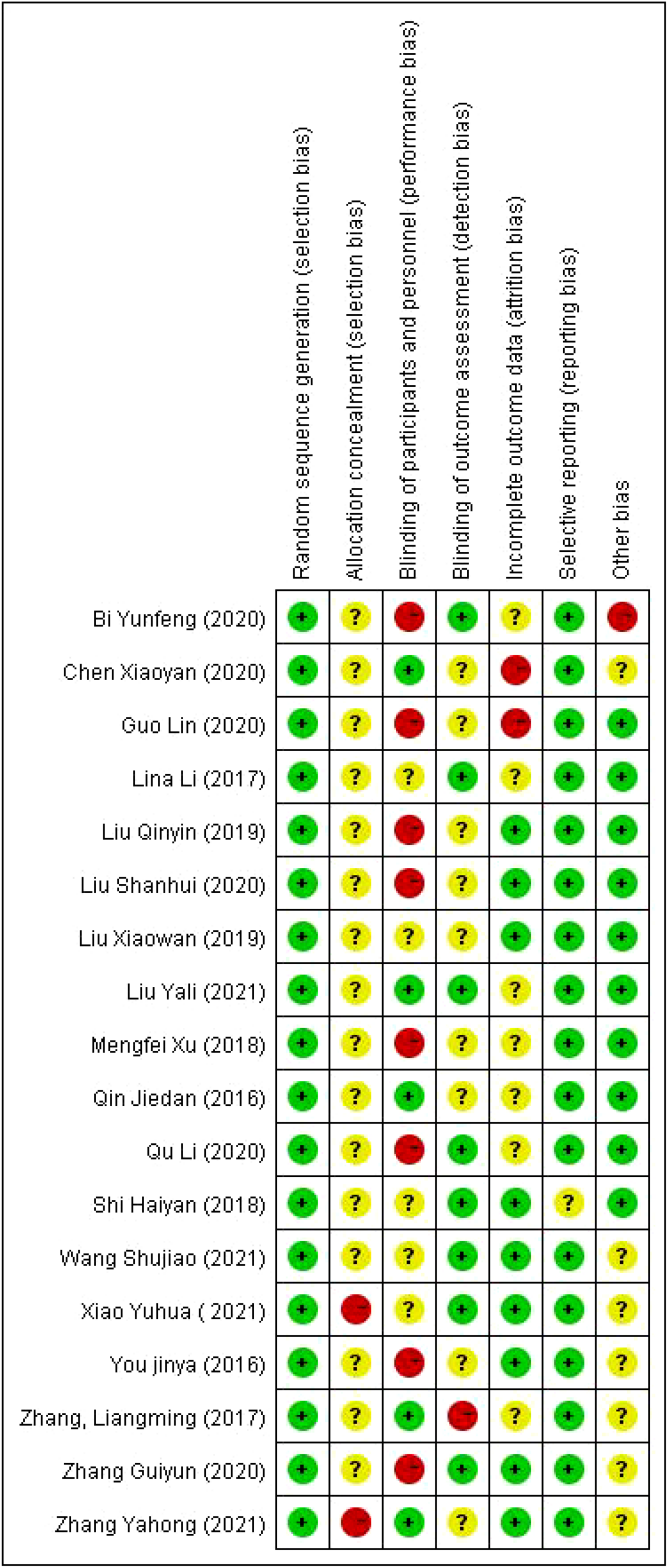
Specific diagram for the evaluation of the quality of sources.

A total of 18 articles were included (16–33). A total of 1773 postoperative patients with cervical cancer were included, and the basic characteristics of the included sources are shown in **Table 1**. The 18 included sources used randomized controlled methods, and the outcome indicators were complete, no selective reporting of results, and no other bias. The quality evaluation of the included sources is shown in **Figures 3**, **4**.

**Table 1:** 18 basic characteristics of the sources.

Author (year)	City	Type of participant	Number of participants			Interventions		Outcomes
			Total	E	C	E	C	
Wang Shujiao (2021) (16)	Hubei	Postoperative patients with cervical cancer	80	40	40	Routine + continuity of care	Routine care measures	Quality of Life Evaluation Scale (SF-36); Self-Assessment Scale for Psychological Emotions Depression (SAS); Self-Assessment Scale for Anxiety (SDS); Satisfaction with Nursing Care
Guo Lin (2020) (17)	Shandong	Postoperative patients with cervical cancer	100	50	50	Routine + continuity of care	Routine care measures	Quality of Life Rating Scale (SF-36); Self-Assessment Scale for Depression (SAS); Self-Assessment Scale for Anxiety (SDS)
Qin Jiedan (2016) (18)	Jiangsu	Postoperative patients with cervical cancer	84	42	42	Routine + continuity of care	Routine care measures	Self-Care Competency Assessment Scale (ESCA); Disease Self-Management Efficacy Measure; Survival Quality Quantity (EROTCQLQ-C30)
Shi Haiyan (2018) (19)	Shaanxi	Post-radical cervical cancer patients	100	50	50	Routine + continuity of care	Routine care measures	Quality of Life Rating Scale (SF-36); Self-Assessment Scale for Depression (SAS); Self-Assessment Scale for Anxiety (SDS)
Chen Xiaoyan (2020) (20)	Sichuan	Cervical cancer patients after extensive hysterectomy	109	55	54	Continuity of care	routine care	Quality of Life Scale; anxiety; satisfaction with care;
Bi Yunfeng (2020) (21)	Guizhou	Cervical cancer patients after extensive hysterectomy	60	30	30	Continuity of care	routine care	Quality of Life Assessment Scale (SF-36)
Liu Xiaowan (2019) (22)	He’nan Mengguzu autonomous county in Qinghai	Cervical cancer patients after extensive hysterectomy	120	60	60	Continuity of care	routine care	Quality of Life Rating Scale (SF-36); Satisfaction with Nursing Care; Self-Assessment Scale for Depression (SAS); Self-Assessment Scale for Anxiety (SDS)
Zhang Yahong (2021) (23)	Shaanxi	Cervical cancer patients after extensive hysterectomy	92	50	42	Routine + continuity of care	Routine care measures	Quality of Life Assessment Scale (SF-36); Olson Marital Quality Questionnaire (Olson)
Qu Li (2020) (24)	Shanghai	Cervical cancer patients after extensive hysterectomy	82	41	41	Routine + continuity of care	Routine care measures	Quality of Life Rating Scale (SF-36); Nursing Satisfaction Score; Self-Assessment Scale for Depression (SAS); Self-Assessment Scale for Anxiety (SDS)
Mengfei Xu (2018) (25)	Jiangsu	Cervical cancer patients after extensive hysterectomy	86	43	43	Routine + continuity of care	Routine care measures	Cancer Rehabilitation Evaluation Scale; satisfaction with care;
Zhang, Liangming (2017) (26)	Hubei	Postoperative patients with cervical cancer	86	48	38	Routine + continuity of care	Routine care measures	Quality of Life QOL Scale; Satisfaction with Nursing Care
Zhang Guiyun (2020) (27)	Liaoning	Postoperative patients with cervical cancer	146	73	73	Routine + continuity of care	Routine care measures	Quality of Life Rating Scale (SF-36); Self-Assessment Scale for Anxiety (SAS)
Xiao Yuhua (2021) (28)	hillsides	Postoperative patients with cervical cancer	92	46	46	Routine + continuity of care	Routine care measures	Survival Quality Measurement Scale WHOQOL-100; Psychological Primacy Scale (RS); satisfaction with care;
Liu Shanhui (2020) (29)	He’nan Mengguzu autonomous county in Qinghai	Patients after radical cervical cancer surgery	80	40	40	Routine + continuity of care	Routine care measures	Self-management Competency Assessment Scale (ESCA); Quality of Life Measurement Scale (QLQ-C30);
Lina Li (2017) (30)	He’nan Mengguzu autonomous county in Qinghai	Postoperative patients with cervical cancer	96	48	48	Routine + continuity of care	Routine care measures	Self-Care Competency Assessment (ESCA); Self-Assessment Scale for Depression (SAS); Self-Assessment Scale for Anxiety (SDS).
Eugenia (2016) (31)	Jiangsu	Postoperative patients with cervical cancer	98	49	49	Routine + continuity of care	Routine care measures	Short form for evaluation of cancer rehabilitation; incidence of postoperative urinary retention and incidence of abdominal distension and incision infection at 6 months after discharge, and time to sexual recovery;
Liu Yali (2021) (32)	Jiangsu	Post-radical cervical cancer patients	82	41	41	Routine + continuity of care	Routine care measures	Quality of life; sleep quality status (PSQI score)
Liu Qinyin (2019) (33)	Hunan	Patients after radical cervical cancer surgery	94	47	47	Routine + continuity of care	Routine care measures	Self-Assessment Scale for Anxiety (SAS), Self-Depression Scale (SDS); WHOQOL-BREF Quality of Life Scale; self-care skills;

### 3.3 Meta-analysis results

#### 3.3.1 Impact of continuity of care on quality of life of patients after cervical cancer surgery

**Figure 5:**
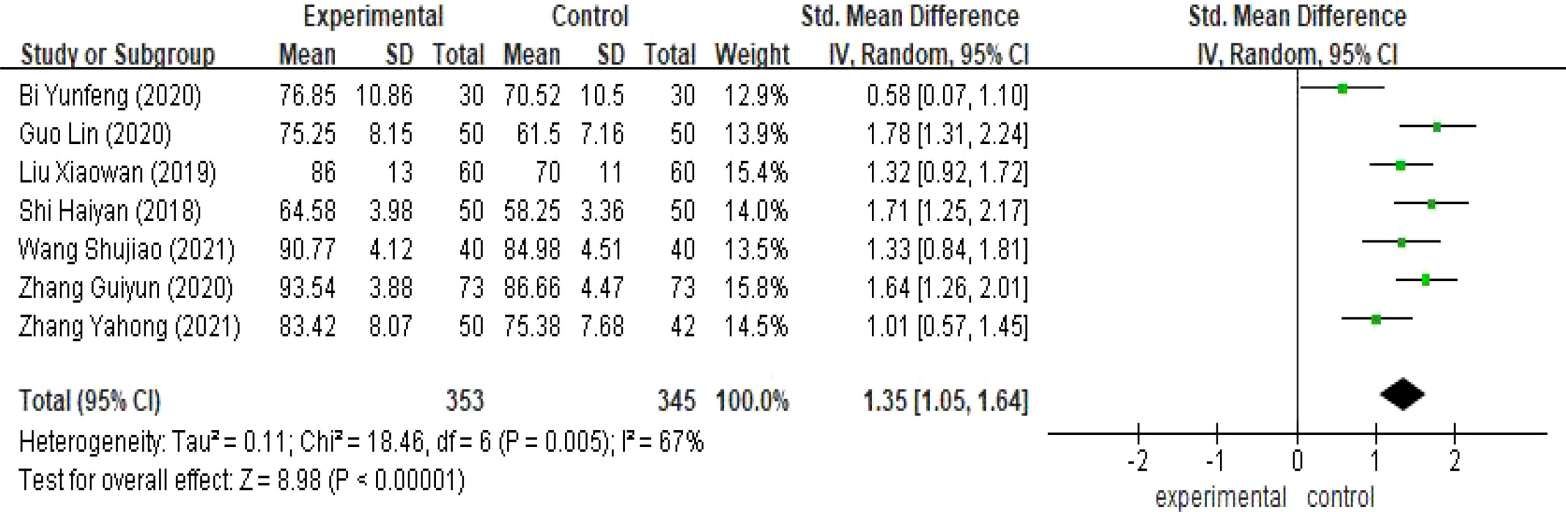
Meta-analysis results of quality of life.

Seven studies (16, 17, 19, 21–23, 27), reported the effect of continuity of care on the quality of life of patients with cervical cancer, seven studies with combinable data were analyzed, and the combined results revealed inter-study heterogeneity (I^2^ = 67%, P<0.001), so Meta-analysis was performed using a random-effects model. The results showed a statistically significant difference [SMD=1.35,95%CI(1.05,1.64),P<0.05], and the results indicated that the quality of survival of cervical cancer patients in the observation group was better than that of the control group (see **Figure 5**).

#### 3.3.2 Effect of continuity of care on anxiety in postoperative cervical cancer patients

**Figure 6:**
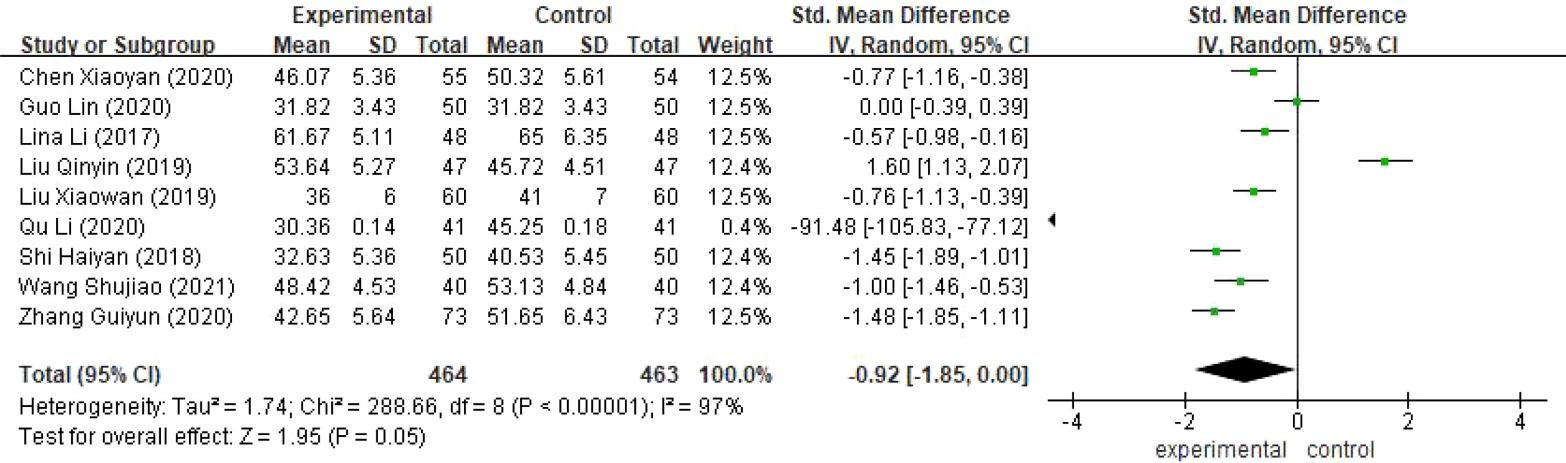
Meta-analysis results of Anxiety Self-Rating Scale (SDS).

There were nine studies (16, 17, 19, 20, 22, 24, 27, 30, 33) comparing the anxiety scores of the two groups of patients with a total of 927 patients with cervical cancer, and because of the large heterogeneity (I^2^ >50%), the results showed that compared with the observation group, the anxiety scores of the control group of patients with cervical cancer were higher, and the difference was statistically significant [SMD=-0.92, 95% CI (-1.85,0.00),P<0.05], as shown in **Figure 6**.

#### 3.3.3 Effect of continuity of care on depression in postoperative cervical cancer patients

**Figure 7:**
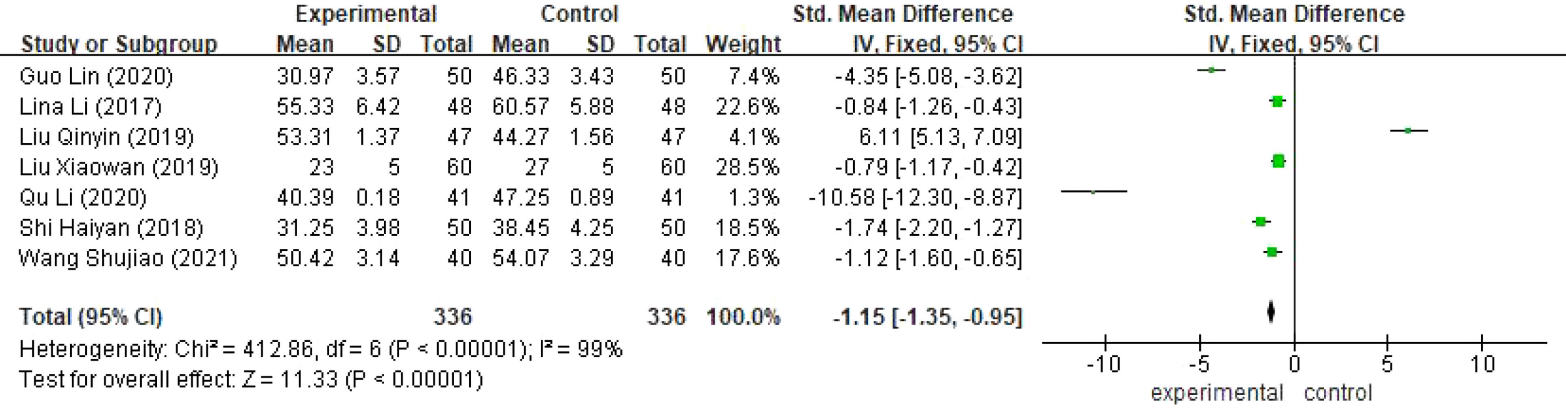
Meta-analysis results of Self-Assessment Scale for Depression (SAS).

Seven studies (16, 17, 19, 22, 24, 30, 33) reported the depression scores of the two groups of 672 cervical cancer patients with large heterogeneity(I^2^ >50%),and the results showed that the difference was statistically significant[SMD=-1.15,95%,CI(-1.35,-0.95),P<0.05],and the implementation of continuity of care, the depression scores of cervical cancer patients in the experimental group were lower than those of the control group, as shown in **Figure 7**.

## 4 Discussion

### 4.1 Continuity of care improves the quality of life of patients after cervical cancer surgery

The findings of this study indicated that there was no significant difference in quality of life between the control group and the intervention group prior to the continuity intervention; however, post-intervention, the quality of life in the intervention group was significantly higher than both their pre-intervention levels and those of the control group (P<0.05). Dietary education: nursing staff should consistently provide targeted and practical guidance to patients based on their evolving conditions and dietary habits. Although most health issues faced by patients are addressed during hospitalization, many individuals continue to experience persistent or new health problems after discharge, resulting in ongoing high healthcare needs among discharged patients (34).

Continuity of care interventions offer patient-centered support that transcends temporal and spatial limitations. The active involvement of families in these interventions effectively enhances postoperative recovery and overall quality of life for patients, facilitating problem-solving at home through direct consultations via internet platforms, telephone communication, and newsletters. This approach not only improves efficiency but also fosters better nurse-patient relationships, achieving a truly zero-distance interaction between nurses and patients. Consequently, it enhances nursing care effectiveness, elevates patient survival quality, increases recognition for nursing work among patients, thereby ensuring effective high-quality care that garners patient affirmation (35).

### 4.2 Continuity of care reduces depression in postoperative cervical cancer patients

After meta-analysis, we found that continuity of care intervention, as a cutting-edge nursing service model, not only enabled patients to have a more comprehensive understanding of cervical cancer, but also provided them with strong support psychologically, and significantly reduced the negative emotion of depression due to the disease (P<0.05). Cancer imposes a high burden of care and also leads to high medical costs, which stresses patients (36). By using the continuity of care model that combines online and offline to help patients gain the knowledge related to cervical cancer, psychologically reduce or eliminate the fear of cervical cancer and depression and other emotional barriers, enhance the patients’ confidence in the recovery of the disease, and more positively face life out of the shadow of the disease, to help the patients to maintain a positive and optimistic mood, and to establish confidence in recovery.

### 4.3 Continuity of care relieves anxiety in postoperative cervical cancer patients

The analysis of anxiety scores revealed that the continuity of care intervention significantly impacted the psychological well-being of cervical cancer patients. Following the implementation of this innovative nursing model, patients exhibited a marked reduction in anxiety levels (P<0.05), indicating positive psychological changes. The continuity nursing intervention emphasizes not only patient care during hospitalization but also ongoing support after discharge. Through regular follow-up visits and meaningful telephone communication, a strong emotional bond was cultivated between healthcare professionals and patients, fostering an environment where patients could experience genuine care and compassion. This continuous support enhances patients’ confidence throughout their recovery journey, enabling them to confront life’s challenges with resilience. Furthermore, as cervical cancer patients typically spend considerable time at home post-discharge, implementing continuity of care can promote psychological comfort and improve mood while alleviating family members’ anxiety related to care giving responsibilities and financial concerns (37). Thus, it is evident that continuity of care interventions provide significant benefits for cervical cancer patients.

## 5 Limitations and perspectives

While this study has made notable strides in examining the impact of continuity of care interventions on cervical cancer patients, several limitations warrant further investigation and enhancement. Firstly, the research predominantly employed scale analysis to evaluate outcome indicators—a method that, despite its widespread use, is inherently susceptible to subjective influences and may introduce bias into the findings. To more accurately capture the effects of interventions, future studies should consider incorporating additional objective and quantitative measures or employing a combination of diverse assessment methodologies to yield more comprehensive and precise data.

Secondly, this study’s reliance on Chinese sources for data collection may have resulted in an oversight regarding significant contributions from relevant international literature and datasets. To expand research perspectives, subsequent investigations should prioritize cross-cultural source collection and analysis to achieve a more holistic understanding of the advancements and applications of continuity of care interventions both domestically and internationally. Furthermore, this study lacks an evaluation of long-term intervention effects due to insufficient follow-up data support. Given that continuity of care is an ongoing process, it is essential to assess its long-term impacts through extended observation periods. Therefore, future research should enhance patient follow-up efforts to gather extensive longitudinal data concerning intervention outcomes, thereby providing robust evidence for clinical practice.

## 6 Conclusion

The results of the study showed that through the implementation of continuity of care, the SF-36 scores of cervical cancer patients were significantly improved, and negative emotions such as anxiety and depression were effectively alleviated, thus substantially improving the quality of life of patients. However, the current continuity of care model has not yet been unified, and the specialized training appears to be insufficient, resulting in a set of clear operational standards that have not yet been formed in the clinic. Therefore, in future studies, we propose to learn from advanced foreign experiences, adopt a multidisciplinary and multi-health care personnel to participate in the model, and form a cross-disciplinary professional team. Through specialized training and guidance, we will ensure that every staff member involved in continuity of care will be able to master the relevant knowledge and skills, and provide comprehensive, continuous, and personalized care services for patients.
